# Intestinal Microbiota Influences DNA Methylome and Susceptibility to Colorectal Cancer

**DOI:** 10.3390/genes11070808

**Published:** 2020-07-16

**Authors:** Aïcha Zouggar, Joshua R. Haebe, Yannick D. Benoit

**Affiliations:** Department of Cellular and Molecular Medicine, University of Ottawa, Ottawa, ON K1H 8M5, Canada; azoug049@uottawa.ca (A.Z.); jhaeb085@uottawa.ca (J.R.H.)

**Keywords:** colorectal cancer, epigenetics, microbiota, DNA methylation, TET3, LINE1

## Abstract

In a recent publication, Ansari et al. identified gut microbiota as a critical mediator of the intestinal inflammatory response through epigenetic programming of host intestinal epithelium. Exposure to the microbiota induces Ten-Eleven-Translocation (TET)-dependent hypomethylation of genomic elements regulating genes associated with inflammatory response and colorectal cancer. Here, we discuss the impact of such a discovery on the understanding of how the intestinal microbiota may contribute to epigenetic reprogramming and influence the onset of colorectal tumorigenesis. Finally, we examine the prospect of TET inhibition strategies as a therapeutic and/or preventive approach for colorectal cancer in patients afflicted by inflammatory bowel disease.

Colorectal cancer (CRC) is one of the most lethal and prevalent cancers globally, likely owing to the complexity of the colonic environment [1]. The human gut microbiota includes trillions of microorganisms playing a fundamental role in regulating the interactions of intestinal epithelial cells with environmental drivers [2]. Important changes in the constitution and metabolome of the microbiota were observed in CRC patients. It is not clear yet whether such changes represent causes or consequences of CRC. Nevertheless, the microbiota plays integral roles in human health and disease, namely promoting the development of a functional immune system, which is expected to play a protective role against CRC [3]. Accordingly, multiple recent studies have been linking antibiotic-induced dysbiosis-alteration in the composition of the intestinal microbiota to increased risks of developing CRC [4,5,6]. In contrast, increasing evidence demonstrates that commensal gut microbiota can also contribute to the development and progression of CRC and its limited response to treatment [4,7,8]. Intestinal microbiota can contribute to a pro-oncogenic colonic environment through chronic inflammation, such as in inflammatory bowel disease (IBD), a common precursor to CRC [9]. The relationship between chronic inflammation and CRC onset was recently shown to be driven, in large part, by epigenetic alterations enhancing the susceptibility of colonic cells to neoplastic transformation [9]. Thus, understanding the interplay between gut commensal bacteria and intestinal cells, at the level of chromatin organization and regulation of specific transcriptional programs, is critical to further characterize CRC etiology.

In a recent publication in Nature Microbiology, Ansari et al. (2020) identified gut microbiota as a critical mediator of the intestinal inflammatory response through epigenetic programming [2]. In this study, the authors use an elegant germ-free (GF) and germ-containing (GC) murine model to contrast the differing DNA methylation signatures resulting from the presence or absence of microbiota. Their findings revealed that colonic crypt cells isolated from GC mice presented significant DNA hypomethylation of active regulatory elements located in low-methylated regions, compared to GF counterparts. The majority of these hypomethylated low-methylated regions were associated with transcriptionally upregulated genes in GC colonic crypt cells compared to the GF group. Such an upregulated gene set was correlated to colitis/IBD and the early inflammatory response in gene ontology analysis. The authors also found a high enrichment of binding sites for FoxA, Eklf and AP1 transcription factors in corresponding hypomethylated low-methylated regions. Those factors were previously linked to gut homeostasis and the inflammatory response [2]. These findings subscribe to the concept of a microbiota-dependent epigenetic landscape in the gut, based on previous genome-wide histone modification analyses showing that commensal bacteria regulate chromatin organization in intestinal immune cell subpopulations [4].

Previous studies have demonstrated that chronic inflammatory signals establish epigenetic silencing of a specific set of genes in colonic epithelial cells, which contribute to inflammation-induced transformation [4]. In turn, Ansari et al. investigated the effect of microbiota on the regulation of acute inflammation using a dextran sodium sulfate (DSS)-based murine model. GC and GF mice were treated with DSS, and whole-genome bisulfite sequencing (WGBS) analyses were performed on colonic crypt cells. While DSS exerted only a minor impact on the low-methylated regions methylation state and associated gene expression in GF mice, GC/DSS mice showed substantial hypomethylation, particularly within lamina-associated domains or genomic regions interacting with the inner nuclear membrane. Hypomethylated genes within lamina-associated domains mainly showed transcriptional up regulation in DSS-treated GC mice compared to controls. Interestingly, lamina-associated domains were previously shown to undergo profound changes to DNA methylation in CRC [10]. Gene ontology analysis revealed that hypomethylated and transcriptionally activated genes in DSS/GC mice (vs. control) were robustly enriched in genes associated with colon cancer. As a final validation for the implication of the intestinal microbiota in such epigenetic reprograming, the authors carried out fecal transplantation experiments to introduce GC gut microbiota into GF mice. This intervention effectively restored DNA methylation to a state resembling GC mice, thereby solidifying the critical role of commensal microbiota in the maintenance of the intestinal epithelial epigenetic signature.

Mechanistically, the authors reported that Ten-Eleven-Translocation 3 (TET3) enzyme expression was significantly up regulated in colonic crypt cells from GC mice vs. GF animals. TET enzymes participate in activating the DNA demethylation process by catalyzing the conversion of 5-methylcytosine to 5-hydroxymethylcytosine [11]. Moreover, antibiotic-induced dysbiosis in GC mice downregulated TET3 expression in colonic cells, while DSS treatment had the opposite effect. Unsurprisingly, WGBS analyses performed on colonic cells from TET2/3 intestine-specific knockout mice (*Tet2/3^fl/fl^* villinCRE) demonstrated a global hypermethylation of low-methylated regions compared to control animals. Specific genes associated with microbiota-induced hypomethylation of low-methylated regions in GC/DSS mice, such as cd177, Pla2g2a and Lpo, were found to be concomitantly hypermethylated and transcriptionally repressed in TET2/3 knockout animals. Ultimately, DSS treatments in TET2/3 knockout mice failed to induce hypomethylation of low-methylated regions associated with microbiota-dependent genes previously identified in GC/DSS animals. This was accompanied by the worsening of clinical scores and impaired inflammatory responses, similar to GF mice. Altogether, Ansari et al. establish a functional relationship between the intestinal microbiota, the expression of active demethylation machinery and chromatin organization in the colonic epithelium (Figure 1). The remodeling of the DNA methylation signature observed in response to pro-inflammatory insults in the presence of microbiota induces transcriptional changes characteristic of CRC.

Interestingly, TET enzymes have previously been implicated in immune cell regulation and colonocyte differentiation [12,13], making their involvement in colonic inflammatory responses in relation to microbial interference no surprise. However, TET family proteins have also been implicated in CRC development, as their dysregulation can alter the epigenetic landscape in colonocytes to promote malignant transformation [12]. TET enzymes were extensively linked to the regulation of pluripotency and self-renewal, two key features of cancer stem cells and hierarchical tumor heterogeneity [14,15]. DNA hypomethylation has also been associated with the increased mobility of transposable elements, which constitute a large proportion of the eukaryotic genome and are maintained inactive by DNA methylation in normal somatic cells [16]. In particular, long interspersed element-1 (LINE1) represents a family of autonomous transposable elements, identified as drivers of tumorigenesis upon demethylation-induced reactivation and insertion/transposition, causing profound transcriptional changes and genomic instability [17]. LINE1 was specifically linked to the maintenance of pluripotency gene networks, tumor initiation events, and the establishment of hierarchical heterogeneity in solid tumors, including CRC [18,19,20,21]. Interestingly, LINE1 transposable elements are enriched in the nuclear periphery and lamina-associated domains, making them potential candidates to be affected by microbiota-induced demethylation and further reactivation [18] (Figure 1). Moreover, TET enzymes were shown to demethylate LINE1 transposable elements in various contexts, including pluripotency and other early phases of development [22]. In particular, TET3-dependent demethylation of transposable elements is specifically involved in epigenetic reprogramming, such as observed in cancer initiation [22,23]. Thus, TET-dependent demethylation of LINE1 elements located in lamina-associated domains, upon colonic cell exposure to microbiota, could represent a first step in pro-oncogenic epigenome reprogramming, facilitating transformation and genome instability.

Collectively, Ansari et al. illuminate the importance of investigations evaluating dysbiosis at the molecular level, with specific regard to DNA methylation, as a causative driver of colorectal carcinogenesis. Critically, the identification of the TET family, specifically TET3, as an invaluable regulator of the inflammatory response in the colon, provides the cornerstone to empirically connecting the health of the commensal bacteria residing in the gut to the development of cancer. Moreover, the recognition of DNA methylation as the mechanism through which the microbiota exerts its effects, establishes the role of the microbiota as a contributing factor to non-hereditary regulation of genes. There are still outstanding questions regarding how commensal bacteria specifically regulate TET expression. Recent data suggest differential impacts from specific metabolites produced by the microbiota on colorectal carcinogenesis. For instance, the short-chain fatty acids acetate, propionate and butyrate display protective effects against CRC, while secondary bile acids promote neoplastic transformation [24]. Thus, the prospect of using molecular messengers produced by the microbiota to modulate TET genes warrants further investigation. Given the relationship of the gut microbiota with the origin and development of CRC as discussed above, there is an increasing interest in exploring microbiome-related therapies for aiding in the prevention and treatment of this cancer. In fact, probiotics or fecal transplantation protocols show promise to combat CRC-associated pro-carcinogenic pathways [25]. In addition, pharmacological approaches targeting TET demethylating activity, such as the small molecule C35, may be beneficial in regulating the inflammatory response and could be explored as a prophylactic approach to prevent CRC, especially in patients suffering from IBD [26]. As such, investigating the effect of new therapeutic strategies for the treatment of CRC, that benefit from the combined strategy of chemotherapy and/or immunotherapy with adjuvant treatments targeting the gut microbiota, could represent elevated clinical potential in personalized medicine [8].

## Figures and Tables

**Figure 1:**
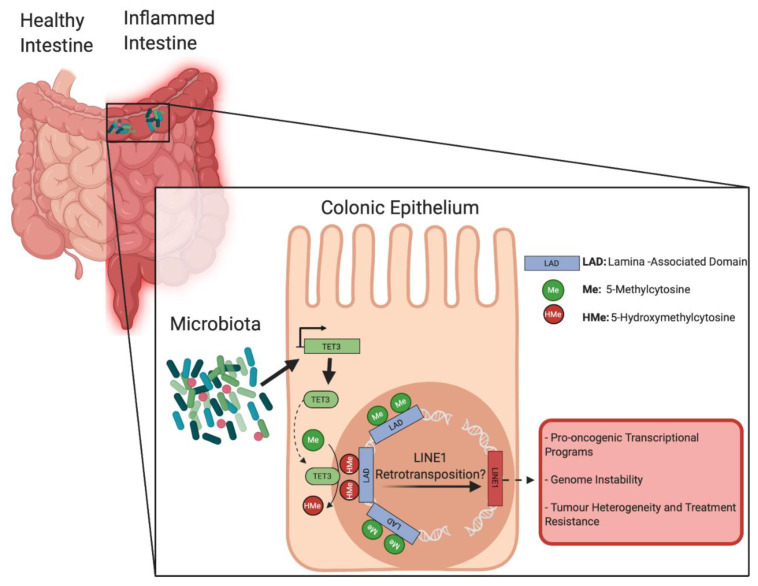
The intestinal microbiota influences DNA methylome and susceptibility to colorectal cancer (CRC). Relationship between the intestinal microbiota and active demethylation machinery in the context of bowel inflammation. Microbiota-induced Ten-Eleven-Translocation 3 (TET3) expression is reorganizing the epigenetic landscape of lamina-associated domains (LADs) in colonocytes, leading to transcriptional changes characteristic of CRC. Image created with BioRender.com.

## Abbreviations

CRC	Colorectal cancer
DSS	Dextran sodium sulfate
GF	Germ-free
GC	Germ-containing
IBD	Inflammatory bowel disease
LINE1	Long interspersed element-1
TET	Ten-Eleven-Translocation
WGBS	Whole-genome bisulfite sequencing
